# Supplementary material for: Adenosinergic System and BDNF Signaling Changes as a Cross-Sectional Feature of RTT: Characterization of Mecp2 Heterozygous Mouse Females
Source: Int J Mol Sci. 2023 Nov 13;24(22):16249. doi: 10.3390/ijms242216249 (PMC10671708; doi:10.3390/ijms242216249)
Supplement: Supplementary file 1 [file ijms-24-16249-s001.zip › ijms-2654762-supplementary.pdf]

Article

The Rett Syndrome Phenotype of Heterozygous *Mecp2* Female Mice Is Associated with a Milder Impact on BDNF/Adenosine Synergism

Catarina Miranda-Lourenço[1], Jéssica Rosa[1], Nádia Rei[1], Rita F. Belo[1], Ana Luísa Lopes[2], Diogo Silva[2], Cátia Vieira[2], Teresa Magalhães-Cardoso[2], Ricardo Viais[1], Paulo Correia-de-Sá[2], Ana M. Sebastião[1] and Maria J. Diógenes[1,*]

[1] Instituto de Farmacologia e Neurociências, Faculdade de Medicina e Instituto de Medicina Molecular – João Lobo Antunes, Universidade de Lisboa, Portugal and Instituto de Medicina Molecular – João Lobo Antunes, Universidade de Lisboa, Portugal;
[2] Laboratório de Farmacologia e Neurobiologia / MedInUP, Instituto de Ciências Biomédicas Abel Salazar – Universidade do Porto (ICBAS-UP), Portugal.
* Correspondence: diogenes@medicina.ulisboa.pt

Supplementary Materials

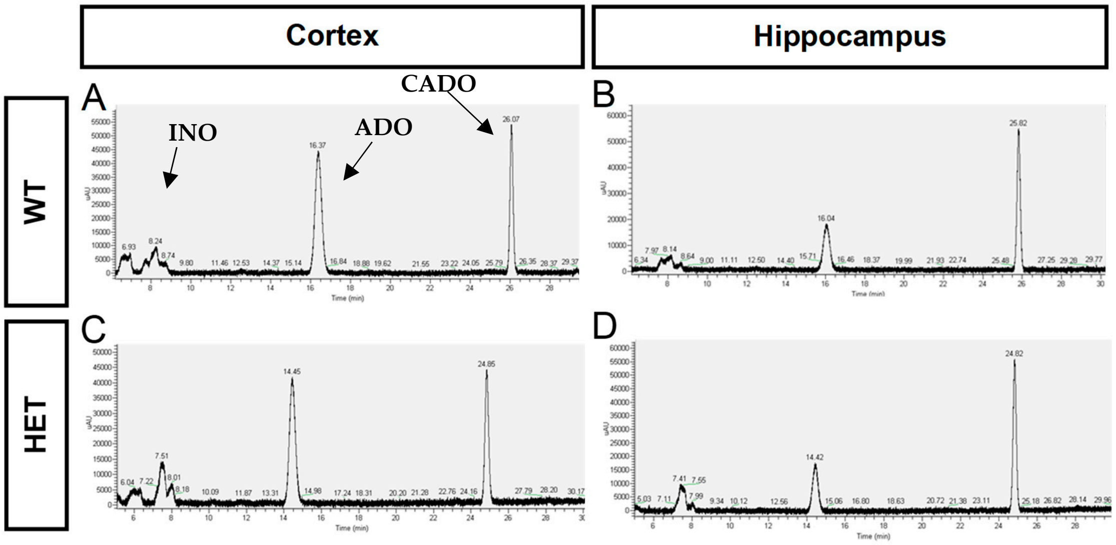

Supplementary Figure S1. Chromatograms obtained from liquid extracts of the cortex (A and C) and hippocampus (B and D) of WT (A and B) and heterozygous Mecp2$^{+/-}$ (C and D) of female mice. Adenine nucleosides were separated by high-performance liquid chromatography with diode array detection (HPLC/DAD), as previously described [13]. The determined elution times for inosine (INO), adenosine (ADO), and 2-chloro-adenosine (CADO, 5 μM—internal standard) were approximately 8, 15, and 25 minutes, respectively (black arrows in Panel A). This method allowed the analysis of all standard liquid tissue extracts with good detection characteristics, 0,157 μM being the lowest and 10 μM the highest concentrations of detected analytes. Calibration curves with a good R^2 value were also achieved for both ADO and INO. DAD-obtained UV spectra confirmed specificity for ADO, INO, and CADO, providing that chosen wavelengths to measure INO and ADO were set to 248 nm and 259 nm, respectively.
